# The Methoxylated, Highly Conjugated C_40_ Carotenoids, Spirilloxanthin and Anhydrorhodovibrin, Can Be Separated Using High Performance Liquid Chromatography with Safe and Environmentally Friendly Solvents

**DOI:** 10.3390/metabo9020020

**Published:** 2019-01-24

**Authors:** Caroline Autenrieth, Robin Ghosh

**Affiliations:** Department of Bioenergetics, Institute of Biomaterials and Biomolecular Systems, University of Stuttgart, Pfaffenwaldring 57, 70569 Stuttgart, Germany; robin.ghosh@bio.uni-stuttgart.de

**Keywords:** carotenoids, HPLC, organic solvents, health protection, safety issues, spirilloxanthin, *Rhodospirillum rubrum*, terpenoids, solvent dielectric constants

## Abstract

High performance liquid chromatography (HPLC) is a frequently used technique in carotenoid research. So far, however, little attention has been paid to the fact that many of the organic solvents used in HPLC separation of highly apolar C_40_ carotenoids impose a significant threat to both health (especially for women) and the general laboratory environment. Here, we developed a solvent combination capable of allowing high-resolution HPLC separation of the C_40_ carotenoid, spirilloxanthin, and all of its biosynthetic precursors beginning with phytoene, using relatively safe, environmentally friendly solvents. We show that separation of spirilloxanthin and its precursors anhydrorhodovibrin and lycopene using modern ultra-high performance chromatography (UHPLC) poses particular problems for apolar carotenoid separation, due to the long residence times in the sample delivery system, which facilitates carotenoid aggregation. We resolved these problems by developing the solvent delivery combination acetone/acetonitrile/isopropanol/methanol (65/30/5/2 (*v/v/v/v*)), which allows excellent column separation using the safe isocratic solvent system methanol/tetrahydrofuran (98/2 (*v/v*)). We also demonstrate that the development strategy for optimizing a solvent system for carotenoid separation can be well-described by the use of the average dielectric constant of the total sample delivery solvent, and present a formal method for analysis of the efficiency of separation.

## 1. Introduction

**Figure 1:**
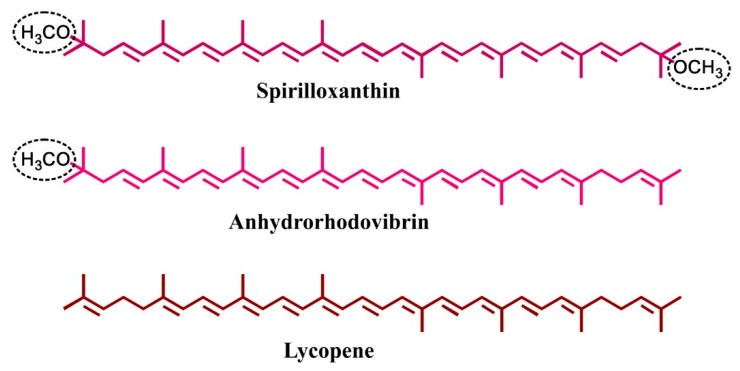
Chemical structures of the C_40_ carotenoids analyzed in this study.

Carotenoids are a colorful class of terpenoids with a vast variety of applications, ranging from food additives to cancer treatment. For instance, lycopene, a linear C_40_ carotenoid containing 11 conjugated double bonds (Figure 1), is used as a food colorant, and may also prevent cancer and cardiovascular diseases (see [1] for a critical review). Additionally, β-carotene, which is synthesized from lycopene by cyclization at both ends of the molecule, serves as precursor of the essential vitamin A. The hydroxylated carotenoids lutein and zeaxanthin are important for vision and cognitive function and have been successfully employed to relieve age-related macular degeneration [2]. Whereas those plant-derived carotenoids contain only 11 conjugated double bonds, which, depending on configuration and concentration, leads to colors from deep yellow to red, several bacterial species can produce deep-purple colored carotenoids with up to 13 conjugated double bonds, such as spirilloxanthin (Spx), a linear C_40_ carotenoid with a methoxy group at both ends of the molecule (Figure 1), which is the wild-type carotenoid found in the photosynthetic membranes of the purple, Gram-negative, non-sulphur bacterium, *Rhodospirillum rubrum*. Even higher conjugation lengths (14–15 double bonds) can be achieved by using in vitro mutagenesis strategies [3,4]. Carotenoids with higher conjugation lengths have been suggested to be more effective anti-oxidants in cancer prevention and treatment than the commonly used carotenoids, such as lycopene [5].

In the above context, Spx is a potentially useful carotenoid for industrial applications. However, in our recent experience in developing methods for large-scale handling of Spx, we have encountered several so-far undocumented problems. The higher conjugation length of Spx imposed challenges for the development of purification methods due to its well-known poor solubility in various commonly used solvents. Perusal of the literature reveals that many of the purification and analytical methods of Spx have been reported for only small quantities of carotenoid, which may mask potential solubility problems. In this study, motivated by the desire to separate Spx by high performance liquid chromatography (HPLC) methods involving relatively safe and environmentally friendly solvents suitable for use in a typical biological laboratory, we present a new solvent combination, which is of general interest for carotenoid analysis.

A comparison of the HPLC methods for the separation of Spx and its precursors (like lycopene) described in the literature, reveals that until the early 1990s, the main solvents used were either methanol (MeOH) [6,7,8,9] or a mixture of MeOH with tetrahydrofuran (THF) (MeOH/THF (98/2 (*v/v*), [10]) on silica-based C_18_ columns, or benzene (containing 1–10% (*v/v*) acetone (Ac)) on Ca(OH)_2_ columns [11,12], or silica gel columns [13]. Subsequently, concurrent with the development of HPLC systems, which utilized high-pressure pumps, as well as the influence of the solvents used for peptide separations using HPLC, other solvent combinations were employed, which contained either mixtures of MeOH and ethylacetate (EtOAc) [11] or acetonitrile (AcN) and EtOAc [14,15]. The use of AcN, which has a very low viscosity, allowed HPLC runs to be performed with higher pressure, i.e., columns with particle sizes smaller than 5 µm could be used, which improved the peak-resolution. AcN was used in combinations with MeOH, isopropanol (iPrOH), THF, or dichloromethane (DCM) [3,5,8,16,17,18,19,20,21]. However, for the separation of Spx all-*E* and *cis* stereoisomers, a more non-polar solvent combination (hexane(Hx)/ethyldiisopropylamine/Ac (98.5/0.05/1.5 (*v/v/v*)) had to be employed [22]. In recent years, parallel to the still successful use of AcN-mixtures [20,21,23], another solvent, methyl *tert*-butyl ether (MTBE), which, like AcN, has a very low viscosity, was employed successfully for the separation of various (mainly plant-derived) carotenoids and apocarotenoids [18,24,25,26,27,28,29,30,31]. Since MTBE is highly non-polar, it has been often employed in gradients in combination with MeOH and H_2_O, in the presence of an ion pair (e.g., ammonium acetate (NH_4_OAc)). However, in the literature, typically high concentrations of MTBE are necessary for the separation of carotenoids, which due to its high vapor pressure (see Discussion), often taxes the ventilation of a typical biological laboratory.

Surprisingly, we found that the effects of solvent properties upon Spx separation were particularly apparent when a modern ultra-high performance liquid chromatography (UHPLC) system was employed, which differs from older systems in that longer delivery times from the autosampler to the separation column occur. Most of the above-mentioned solvent systems have been developed in organic chemistry laboratories and are unsuitable for use in the facilities of a typical biological laboratory, because many of their components (e.g., MTBE, DCM) are generally highly volatile and also very toxic, as well as being harmful for the environment. In 2013, Bóna-Lovász et al. [32] developed a UHPLC solvent system, based only on the less harmful solvent Ac, combined with H_2_O. However, the use of Ac obstructs UV-detection during the HPLC run, and the relatively high water content (nearly 30% at the start of the gradient) led to solubility problems with samples containing lipids. Therefore, in this study, we re-focused on the use of MeOH as the main solvent for the HPLC-method (MeOH/THF (98/2 (*v/v*))), which, in previous studies [4,10] had been shown to yield an excellent separation of Spx and all of the precursors starting from phytoene. The method had to be adapted for its use in an UHPLC system. In particular, a special solvent combination is necessary to ensure effective sample delivery from the sample vial to the column. This UHPLC solvent system is optimal for Spx, and uses solvents which are easily handled and are environmentally friendly. Since all organic solvents show some degree of toxicity, we have focused on those which are most compatible with a typical biology laboratory environment. 

We also show that the HPLC chromatographic behavior of Spx in many well-known solvents is anomalous, compared to that of well-studied C_40_ carotenoids, such as lycopene and β-carotene, which leads to a rapid deterioration in attainable resolution. Finally, we present a possible physical rationale for the optimization of separation solvent efficacy. 

## 2. Results

### 2.1. Initial Considerations for the Separation of Spx Using a Modern UHPLC-System

It has been shown previously that Spx can be easily separated from its biosynthetic precursors (such as lycopene and anhydrorhodovibrin (Anhdrvbr) (Figure 1)) by using a simple isocratic HPLC method (on a BioRad^®^ system) with an ODS-2 (C_18_) column and MeOH/THF (98/2 (*v/v*)) as running solvent [4,10]. In the present study, we initially wanted to reproduce this data by using a modern UHPLC-system (Dionex^®^ Ultimate 3000) and a new C_18_ column (for details, see Materials and Methods section). Both in the BioRad^®^ HPLC system and in the Dionex^®^ UHPLC system, we did not use the running solvent (MeOH/THF (98/2 (*v/v*))) for solubilization of the Spx sample, because Spx is poorly soluble in MeOH. This is contradictory to the common recommendation that the sample should be solubilized in the running solvent. In this study, we show that optimization of both the *Sample Solvent*, which is used to solubilize the Spx sample, and the *Syringe Solvent*, which delivers the sample to the column (see below), is necessary for optimal UHPLC separation. In contrast, the solvent system, which is pumped through the column isocratically and used for chromatographic separation (the *Running Solvent* (MeOH/THF (98/2 (*v/v*))), was kept constant.

**Figure 2:**
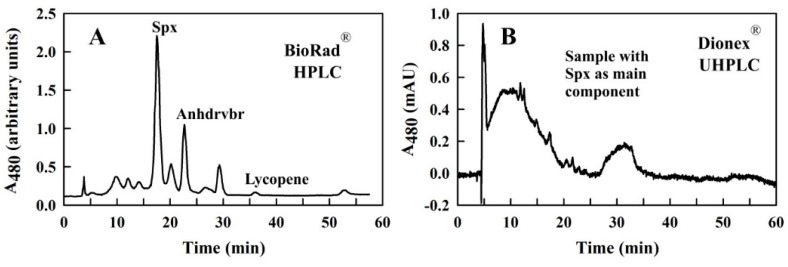
Comparison of HPLC runs with (**A**) the BioRad^®^ HPLC system [4], and (**B**) the Dionex^®^ UHPLC system. In both cases, a sample containing Spx as main component, and with minor components Anhdrvbr and lycopene, was injected. The *Running Solvent* (and, for (**B**), the *Syringe Solvent*) was MeOH/THF (98/2 (*v/v*)) (with a flow-rate of 0.5 mL/min), and the *Sample Solvent* was Hx.

**Figure 3:**
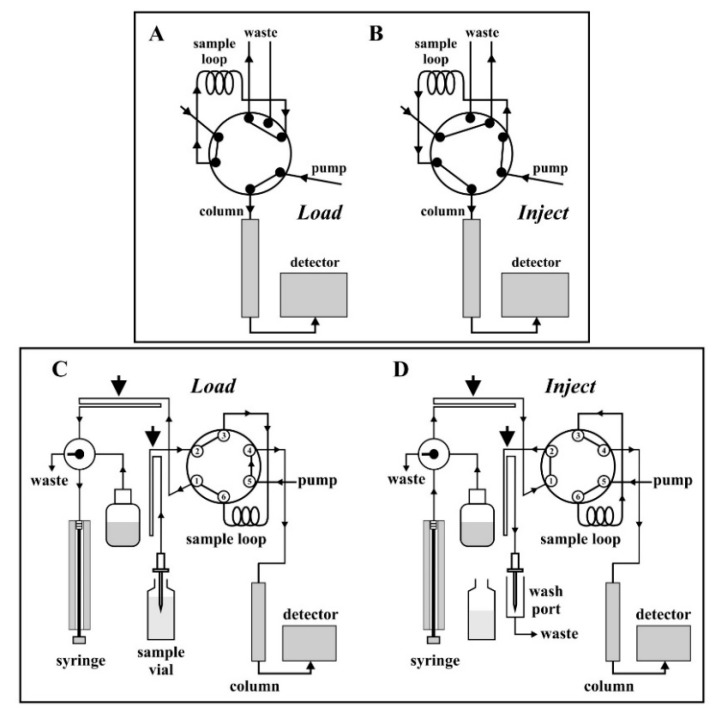
Comparison of the injection setups of the BioRad^®^ HPLC (**A**,**B**) and the Dionex^®^ UHPLC (**C**,**D**) systems. In (**A**,**C**) the “load” position and in (**B**,**D**) the “inject” position is shown, respectively. (**C**,**D**) were drawn with the program CorelDRAW X8^®^ using information from the Dionex^®^ autosampler ACC-3000T instruction manual. The large arrows in (**C**,**D**) indicate the long tubing connections, which are filled with the *Syringe Solvent*.

In a first set of experiments, Hx was used as *Sample Solvent* (because it had been used in the initial purification steps). Surprisingly, in contrast to the HPLC runs with the BioRad^®^ system (Figure 2A), those obtained with the UHPLC system were not reproducible and did not show any resolution of defined peaks, but only a poorly resolved broad band (Figure 2B). We deduced that the reason for the observed differences in profiles for the two systems may have its origin in the physical delivery of the sample to the column. A comparison of the injection setups in both the HPLC and UHPLC systems, respectively, is shown in Figure 3. In the BioRad^®^ system, the distance between the manual injection point and the sample loop is very short (the needle port is located on the injection valve), as is the connection between the sample loop and the column (Figure 3A,B). In the UHPLC system, the respective distances are much longer (Figure 3C,D). In the UHPLC loading process (Figure 3C), the movement of liquid from the sample vial of the autosampler requires the autosampler needle and tubing pathway to the sample loop to be pre-filled with a syringe solvent (the *Syringe Solvent*) before sampling can occur. 

In the first set of experiments, the *Syringe Solvent* was the same as the *Running Solvent* (MeOH/THF (98/2 (*v/v*))). However, we realized, that in the UHPLC system, the much longer path of the *Syringe Solvent* tubing system led to inhomogeneous mixing with the *Sample Solvent* Hx, due to its poor solubility in MeOH. An additional problem was the high volatility of Hx, which caused evaporation during the waiting time of the loading process, with subsequent clotting of the sample needle. Thus, many HPLC runs yielded no signal. 

The inject mode also exhibited two potential residence time problems associated with the instrument configuration. First, the residence time in the sample loop prior to column injection (determined by the software-controlled “pump stroke inject valve synchronization”) is long (tens of seconds), and can lead to inhomogeneous mixing. Secondly, additional mixing problems can occur due to the long residence time between the sample loop and column, since the connecting tubing (30 cm) passes through the column oven in order to thermally equilibrate the solvent.

**Figure 4:**
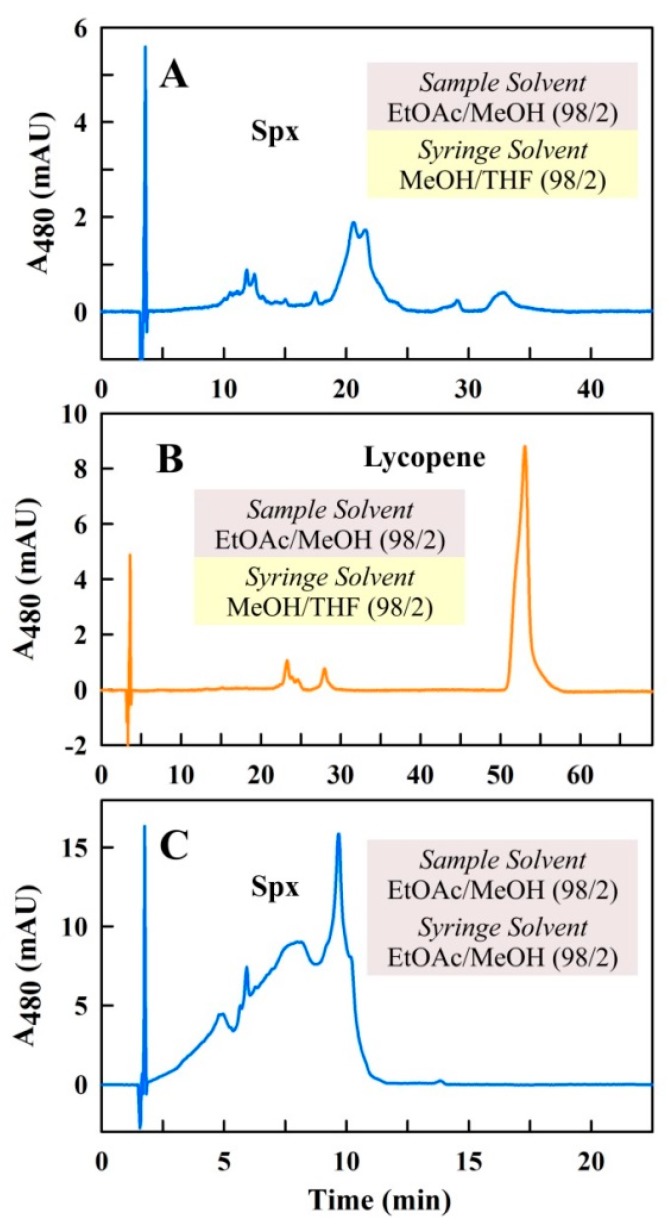
UHPLC runs with Spx-containing samples (**A**,**C**) and a lycopene-containing sample (**B**). The *Running Solvent* was always MeOH/THF (98/2 (*v/v*)) and the *Sample Solvent* was EtOAc/MeOH (98/2 (*v/v*)). In (**A**,**B**), runs with MeOH/THF (98/2 (*v/v*)) as *Syringe Solvent* are shown; in (**C**) the *Syringe Solvent* was EtOAc/MeOH (98/2 (*v/v*)). The flow rates were 0.5 mL/min (**A**,**B**), and 1.0 mL/min (**C**), respectively.

In a second set of experiments, another *Sample Solvent*, EtOAc/MeOH (98/2 (*v/v*)), which was intended to obviate the above-mentioned problems, was employed. EtOAc, which is a good solvent for Spx, has a lower vapor pressure than Hx and mixes readily with the *Syringe* and *Running Solvent* MeOH/THF (98/2 (*v/v*)). To prevent needle clotting by the Spx sample, a longer syringe wash program was used immediately after every injection. Furthermore, between every two consecutive HPLC sample analyses, 20 µL of *Sample Solvent* (EtOAc/MeOH (98/2 (*v/v*))) were injected to clean the needle and system. The HPLC runs obtained with this solvent configuration (Figure 4A) were somewhat better than those with Hx as *Sample Solvent*, i.e., some peaks were distinguishable. Nevertheless, the peaks were still broad and the detected absorbance was much too low compared to the sample concentration employed in this run. Interestingly, injection of a lycopene-containing sample, dissolved in the *Sample Solvent* EtOAc/MeOH (98/2 (*v/v*)), using the same protocol, resulted in reproducible HPLC runs with a sharp peak due to lycopene, where the peak height corresponded well with the concentration employed (Figure 4B). 

The low resolution of the Spx HPLC runs indicated that a further factor must be important. In particular, not only the mixing behavior of the *Sample* and *Syringe Solvent* has to be considered, but also the solubility of the carotenoid (Spx or lycopene, respectively) in the respective solvent systems. Whereas lycopene shows good solubility in MeOH (the main component in both the *Syringe* and *Running Solvent*), this is not the case for Spx, which has an extremely low solubility in that solvent. Possibly, the lower solubility may lead to precipitation of Spx during its passage through the long tubing path (see Figure 3C) between needle, sample loop, and column, all of which are filled with the *Syringe* and *Running Solvent* MeOH/THF (98/2 (*v/v*)).

To evaluate this hypothesis, the *Syringe Solvent* was changed—the syringe and tubing were filled with EtOAc/MeOH (98/2 (*v/v*)) (the same as the *Sample Solvent*), in which Spx exhibits higher solubility than in MeOH/THF (98/2 (*v/v*)). In the event, the total Spx sample was delivered effectively to the column, which can be seen from the higher absorbance values in the HPLC run shown in Figure 4C. In this latter protocol, the tubing between sample loop and column was still filled with the *Running Solvent*. However, this did not have any negative effect upon the HPLC run performance, as evidenced by the observation that transient filling of the tubing (via a gradient program) with high THF concentrations (which should enhance Spx solubilization) prior to injection, followed by a steep negative gradient to restore the *Running Solvent* (MeOH/THF (98/2 (*v/v*)) showed no further improvement in peak resolution. 

In summary, reproducible UHPLC runs require that the *Sample Solvent* is of low volatility (to prevent Spx precipitation in the needle), and that the *Sample* and *Syringe Solvents* are chosen to ensure that Spx remains soluble during the subsequent transport to the column. However, it is not obligatory that both solvents are identical, merely that they are compatible in their mixing properties without having deleterious effects upon Spx solubility. 

### 2.2. Optimization of the Solvent System for Use as Sample and Syringe Solvent for Spx UHPLC Runs

Thin-layer chromatography (TLC) analysis of the Spx sample used indicated that only one carotenoid predominates in the sample. In contrast, the UHPLC profile shown in Figure 4C, using EtOAc/MeOH (98/2 (*v/v*)) as *Sample* and *Syringe Solvent*, exhibited a complex profile, indicating that the solvent composition is not optimal for Spx analysis, leading to aggregation events during the passage between sample vial and column. Therefore, in order to find a better solvent system, different solvent combinations (commonly used as *Running Solvents* in published protocols (see Introduction)) were used as *Sample* and *Syringe Solvent*. In contrast to the published protocols mentioned above, we maintained MeOH/THF (98/2(*v/v*)) as the *Running Solvent*.

**Figure 5:**
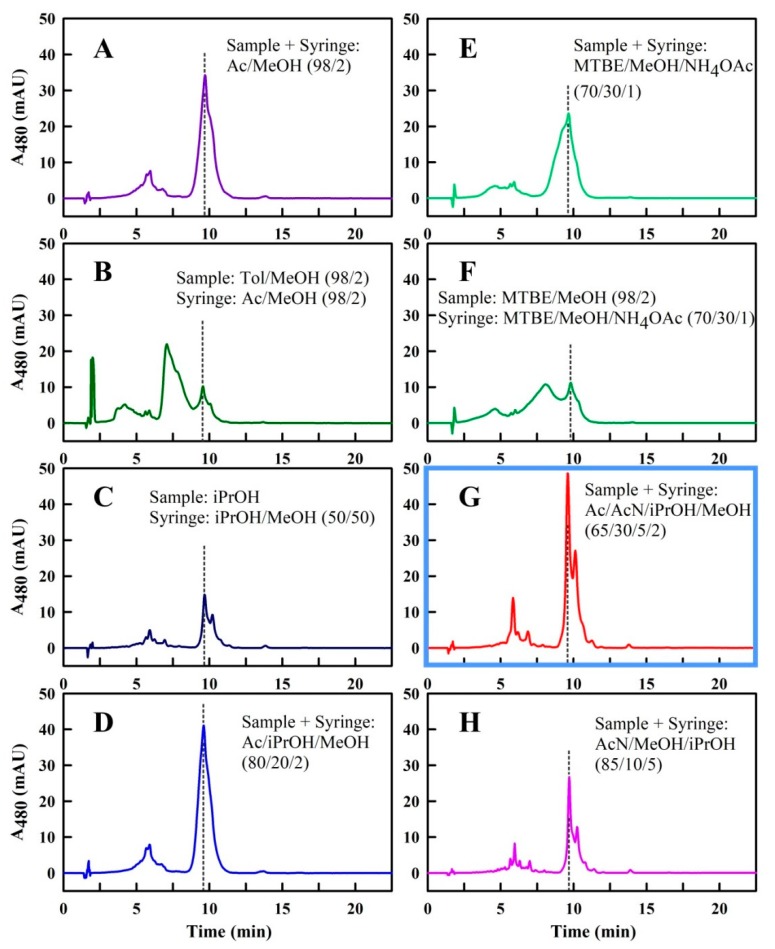
Spx-UHPLC runs. The *Running Solvent* was always MeOH/THF (98/2 (*v/v*)), the flow rate was 1.0 mL/min, and the *Sample* and *Syringe Solvent* were varied (as indicated in the panels (**A**–**H**)). The dashed lines indicate the position of the desired all-*trans* Spx peak (A_max1_), respectively. The optimal run (**G**) is highlighted. Abbreviation not given in the main text: toluene, Tol.

The experiments for finding the optimal solvent combination are shown in Figure 5. When Ac/MeOH (98/2 (*v/v*)), which is known to be a good solvent for Spx [32], was used as *Sample* and *Syringe Solvent*, the peak resolution improved significantly (Figure 5A). In the event, a major peak at approximately 10 min was observed, which could be shown by measurement of the absorption spectrum (during the UHPLC run) to be due to Spx. The smaller, broader peaks around 6 min showed the spectral features of a Spx precursor with 12 conjugated double bonds (presumably rhodovibrin).

Unexpectedly, the use of toluene, which, like Ac, is commonly seen as very good solvent for organic compounds such as carotenoids, yielded an UHPLC profile, showing poor resolution (Figure 5B) for the *Sample Solvent* combination toluene/MeOH (98/2 (*v/v*)). In particular, a large proportion of the Spx sample eluted at retention times around 7 min, which was 3 min before the expected retention time for Spx. The broad peak between 7 and 9 min indicated that Spx had not been well-dissolved when it reached the column, and thus could not interact properly with the C_18_ matrix of the HPLC column. Compared to Ac, toluene is much more apolar (with a dielectric constant (D) of 2.4, compared to D = 20.7 for Ac). Therefore, another *Sample Solvent*, iPrOH, with a polarity (D = 18.3) comparable to Ac, was used in a further trial (Figure 5C). Here, a sharpening, as well as a splitting (due to the all-*trans* and *cis* isomers of Spx, as revealed by spectrum measurement of the peaks during the UHPLC run) of the peak at 10 min could be observed. However, iPrOH was still not the optimal *Sample Solvent*, since the signal was quite low, because the Spx sample had been only poorly soluble in iPrOH (much of the sample was sticking to the wall of the reaction tube after mixing with iPrOH, and could not be solubilized). Thus, in the next trial, we combined the positive effects of Ac and iPrOH, by using the *Sample* and *Syringe Solvent* Ac/iPrOH/MeOH (80/20/2 (*v/v/v*)) (Figure 5D). In this solvent, the Spx sample was well-solubilized and the peak at 10 min was sharper than without the use of iPrOH (compare to Figure 5A). However, the splitting of the Spx peak could no longer be observed.

We also tried a completely different solvent, MTBE, which has been used extensively in carotenoid HPLC analysis [18,24,25,26,27,28,29,30,31]. However, in a run with the *Sample* and *Syringe Solvent* MTBE/MeOH/NH_4_OAc (25 mM) (70/30/1 (*v/v/v*)) (see [24]), we observed a considerable broadening of the Spx peak at 10 min (Figure 5E). Higher MTBE concentrations in the *Sample Solvent* MTBE/MeOH (98/2 (*v/v*)) worsened UHPLC run performance (Figure 5F) even further, and led to the appearance of the broad peak at around 8 min, which had also been observed in the trials with toluene. Thus, MTBE was not a good solvent for the separation of Spx and its biosynthetic precursors.

So far, solvent mixtures with Ac as the main component led to the highest resolution in the initial set of experiments. In a further set of experiments, different combinations of Ac with the widely-used solvent, AcN, were employed. In the event, the combination Ac/AcN/iPrOH/MeOH (65/30/5/2 (*v/v/v/v*)) for both the *Sample* and *Syringe Solvent* resulted in the best UHPLC runs achievable (Figure 5G), with a sharp peak at 9.6 min and resolution of the all-*trans* and *cis* peaks. Routinely, we employed 5% (*v/v*) iPrOH in order to ensure that any traces of contaminant protein in the sample would be soluble and not precipitate in the HPLC system. The low iPrOH concentration had no effect upon the attainable peak resolution. 

Higher AcN concentrations (e.g., AcN/MeOH/iPrOH (85/10/5 (*v/v/v*)), as in [5,8]), resulted in further peak sharpening (Figure 5H). However, as observed also for iPrOH alone, this solvent combination did not solubilize the Spx sample completely, resulting in a considerable sample loss. We therefore consider the *Sample* and *Syringe Solvent* combination Ac/AcN/iPrOH/MeOH (65/30/5/2 (*v/v/v/v*)) (Figure 5G) to be optimal for the separation of Spx in the UHPLC setup.

**Figure 6:**
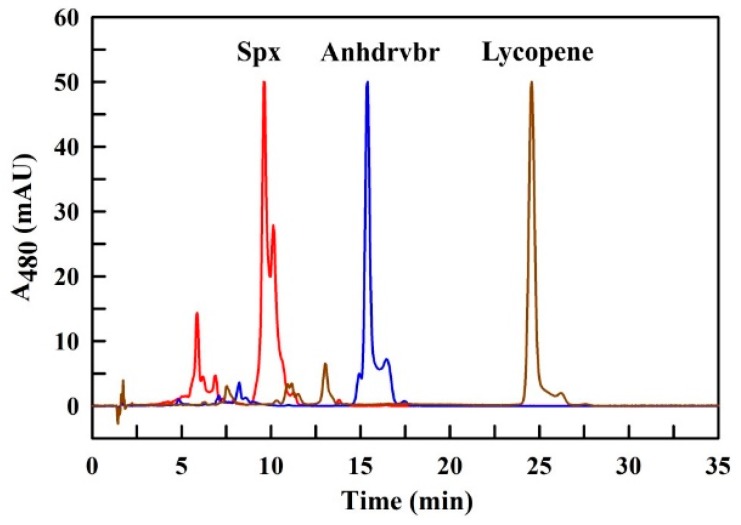
Overlay of three UHPLC runs with the Spx- (red), Anhdrvbr- (blue), and the lycopene- (brown) containing sample, respectively, performed with the optimal *Sample* and *Syringe Solvent* Ac/AcN/iPrOH/MeOH (65/30/5/2 (*v/v/v/v*)). The Spx run is the same as the run shown in Figure 5G. The A_480_ values were normalized to the same peak height at the major peak.

The optimal solvent system obtained here was also used to re-analyze the lycopene-containing sample and also a sample containing Anhdrvbr, a Spx precursor, which contains 12 conjugated double bonds and only a single methoxy end group (see Figure 1). The overlay of the three respective UHPLC runs (Figure 6) clearly shows that those compounds can be well-separated by the UHPLC solvent system developed here. Also, the resolution and sharpness of the peaks have improved when compared to the peak profiles obtained with the BioRad^®^ HPLC system (see Figure 2A).

## 3. Discussion

In this study, our primary goal was to establish conditions for the UHPLC separation of the end-methoxylated C_40_ carotenoid, Spx, as well as all of its biosynthetic precursors starting from phytoene, using relatively safe and cheap solvents, which are also suited to the environment of a typical biology laboratory. Our initial intention was to utilize the isocratic MeOH/THF solvent for separation, which Schwerzmann and Bachofen (1989) [10] showed previously to be excellent for separating all the major carotenoids present in the Spx pathway. However, we were surprised by the fact that the application of this solvent system in a modern UHPLC system led to poor resolution and many spurious peaks. The solution of this problem, which we now understand to reside in the solubility properties of Spx in the sample delivery system (*Sample* and *Syringe*), has led us to a deeper understanding of the underlying principles involved, which to our knowledge have not been reported in the literature so far. We now recognize that the poor peak resolution in the modern system is due to the formation of metastable but soluble aggregates of Spx (and some derivatives) in the sample delivery system, and does not arise principally from incompatibility between carotenoids and mobile phase of the separation column. In particular, we have shown that the use of MeOH as a *Running Solvent* is only possible if an optimal delivery of the sample from the sample vial to the column is ensured. This task is achieved by choosing an optimal solvent combination for both the *Sample* as well as the *Syringe Solvent*. Here, we developed a special solvent combination, which contains acetone and acetonitrile as main components (Ac/AcN/iPrOH/MeOH (65/30/5/2 (*v/v/v/v*))) as *Sample* and *Syringe Solvent* for solubilization and delivery of Spx to the column. This solvent system was initially developed by visual inspection and comparison of various UHPLC runs (see Figure 4 and Figure 5). Although the path to the final solvent system was, to some extent, discovered by trial and error, we have also employed a new semi-quantitative analysis of the separations obtained in various solvent combinations in order to deduce possible underlying principles. 

### 3.1. Quantitative Analysis of the Solvent Delivery System

**Figure 7:**
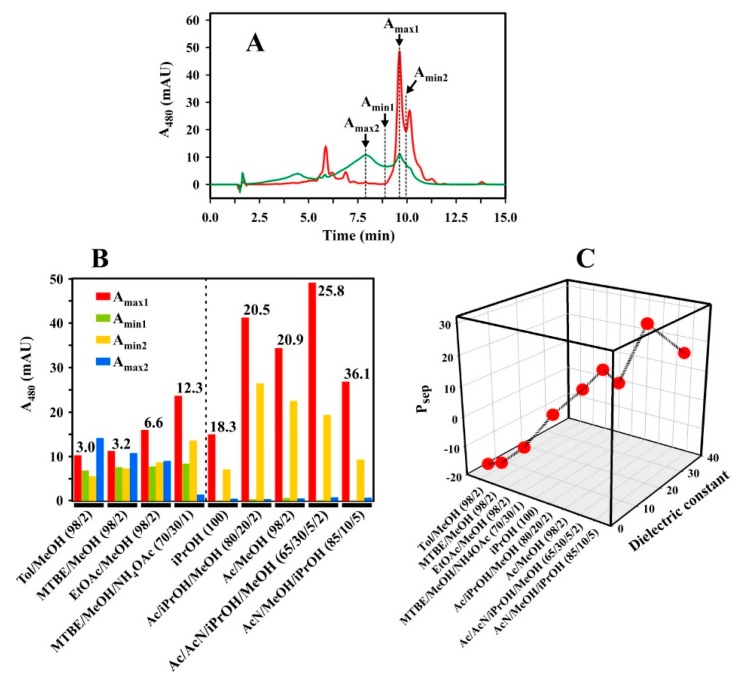
Semi-quantitative analysis of the UHPLC runs. (**A**) The optimal run (red, *Sample* and *Syringe Solvent* Ac/AcN/iPrOH/MeOH (65/30/5/2 (*v/v/v/v*), see Figure 5G) is overlaid with a non-optimal run (green, *Sample Solvent* MTBE/MeOH (98/2 (*v/v*)), *Syringe Solvent* MTBE/MeOH/NH_4_OAc (70/30/1 (*v/v/v*)), shifted by Δ = −0.2 min, compared to the run shown in Figure 5F). The positions of A_max1_, A_max2_, A_min1_, and A_min2_ are indicated. (**B**) The A_max1_, A_max2_, A_min1_, and A_min2_ values of the runs shown in Figure 4C and Figure 5. The runs are sorted according to the respective average dielectric constant (D_av_) values (indicated above each solvent combination) of the *Sample Solvents* used (indicated in the Figure). (**C**) Calculated P_sep_ values for the values shown in (B), plotted against their respective D_av_ values.

For our analysis, we assume that all of the observed peaks in a given solvent system correspond to different molecular states of Spx. Thus, for the formal definition of solvent optimality, we employ the following assumptions (see also Figure 7A):

(1) The solvent delivery system Ac/AcN/iPrOH/MeOH (65/30/5/2 (*v/v/v/v*)) (Figure 5G) is optimal, and the peak (A_max1_) observed at **9.6 min** corresponds exclusively to all-*trans* Spx. In the optimal separation, Spx peaks do not occur elsewhere, and other peaks correspond to other carotenoids present in the same sample (confirmed independently by TLC and spectral measurement during the UHPLC run).

(2) In a non-optimal system, the A_max1_ Spx peak intensity is reduced, and appears correspondingly at other (earlier) times, due to Spx aggregate formation in the sample delivery system. 

(3) Characteristically, Spx aggregate formation is often characterized by peak(s) at the position 7.9 min. We define the intensity at this point as A_max2_.

(4) We have also chosen to include the minimum (A_min1_) occurring at 8.5 min, immediately prior to the all-*trans* Spx peak in the optimal separation, since some aggregated forms also yield intensity at this position. In the optimal separation solvent, this value is essentially zero.

(5) Finally, the optimal separation between all-*trans* and *cis* Spx peaks at 9.6 min and 10.1 min, respectively, can be quantified by measuring the intensity at the minimum (A_min2_) separating the two peaks at 10.0 min. Thus, well-separated all-*trans* and *cis* peaks show a larger (A_max1_-A_min2_) value than those which are poorly separated.

We note that different HPLC runs may show a minimal time shift (Δ) with respect to the optimal profile used to define the parameters A_max1_, A_max2_, A_min1_, A_min2_. In these cases, the time point for parameter estimation was shifted from the above values by a value Δ, with respect to the observed peak maximum for all-*trans* Spx in that profile. For example, in Figure 5A, the Spx peak was observed at 9.7 min (yielding Δ = +0.1 min). In this case, the time points for parameter estimation were 8.0 min, 8.6 min, and 10.1 min for A_max2_, A_min1_, and A_min2_, respectively.

Using this formalism, the performance (P_sep_) of a solvent system can be estimated by the difference equation:

P_sep_ = A_max1_ − (A_max2_ + A_min1_ + A_min2_) (1)

In an optimal separation, the parameters in parentheses take their minimal values, and the value of P_sep_ is maximal.

Since we can safely assume that the spurious peaks in non-optimal profiles are due to the solubility of Spx in the solvents examined, we explored the possibility that solvent optimality may be related to the *average* dielectric constant (D_av_, see Materials and Methods) of the solvent mixture, which reflects its average polarity. Indeed, when the UHPLC runs were sorted corresponding to the D_av_ of the *Sample Solvent* (Figure 7B), some systematic changes of A_max1_, A_min1_, A_min2_, and A_max2_, could be observed, indicating an underlying separation principle. In particular, the runs can be divided into two groups: (a) below an D_av_ of 18, relatively high A_max2_ and A_min1_ values were observed, indicating that a significant portion of Spx is aggregated in those solvents. Correspondingly, the A_max1_ values are quite low in this group; (b) above an D_av_ of 18, A_max2_ and A_min1_ become negligible, and the A_max1_ values, corresponding to the all-*trans* Spx peak, begin to rise to a maximum at D_av_ = 25.8 for the optimal solvent system Ac/AcN/iPrOH/MeOH (65/30/5/2 (*v/v/v/v*)).

When the P_sep_ values are calculated for each solvent, the correlation between P_sep_ and D_av_ is striking (Figure 7C). Thus, we had expected that the Spx molecule, which is composed of a long isoprenoid backbone and a π-electron system extending over 13 conjugated double bonds, should be highly apolar, and therefore well-soluble in a solvent of low dielectric constant. The methoxy groups at both ends of the molecule should be able to interact with solvents, which can act as H-donors in H-bonding systems. However, the very apolar solvent combinations, i.e., those with the lowest D_av_ values, and which are generally considered to be good solvents, like Hx (D = 1.9), toluene/MeOH (98/2 (*v/v*), D_av_ = 3.0), MTBE/MeOH (98/2 (*v/v*), D_av_ = 3.2), chloroform/MeOH (98/2 (*v/v*), D_av_ = 5.4), and EtOAc/MeOH (98/2 (*v/v*), D_av_ = 6.6), yielded negative P_sep_ values when they were used as *Sample Solvents*. The UHPLC runs showed broad peaks and poor resolution. In the case of toluene/MeOH (98/2 (*v/v*)), MTBE/MeOH (98/2 (*v/v*)), and also EtOAc/MeOH (98/2 (*v/v*)), a considerable A_max2_ peak with a 2–3 min shorter retention time than that expected for Spx appeared, respectively, which we consider to be due to aggregation effects in those solvents. This result is counter-intuitive, since we had expected that the methyl and methoxy groups of those solvents should interact with the methoxy groups of Spx, and therefore be good solvents for that compound.

The more polar solvent combination MTBE/MeOH/NH_4_OAc (25 mM) (70/30/1 (*v/v/v*), D_av_ = 12.3) showed a higher P_sep_ (= 0.2) and a corresponding improvement in resolution (Figure 5E), although the Spx peak was still broad. The solvent with an even higher D = 18.3, iPrOH, led to a good separation of the all-*trans* and *cis* Spx peaks (with a positive P_sep_ = 7.2), albeit with a loss of solubility of Spx in this *Sample Solvent*. This problem was overcome by adding 80% (*v/v*) Ac to the solvent mixture (Figure 5D), which raised D_av_ to a value of 20.5 and P_sep_ to 13.9.

So far, raising the D_av_ of the solvent mixture used as *Sample Solvent* had yielded UHPLC runs with a better peak resolution, which is reflected by a concomitant rise in the resulting P_sep_ values. The first outlier in this series is the experiment with Ac/MeOH (98/2 (*v/v*)) as *Sample* and *Syringe Solvent* (Figure 5A, P_sep_ = 10.6), which has a slightly higher D_av_ (20.9) than the solvent combination containing 20% (*v/v*) iPrOH. Thus, iPrOH seems to affect the solubility properties of Spx in a more sophisticated way than by simply changing the polarity of the solvent system. A similar observation can be made if AcN is added to the *Sample* and *Syringe Solvent*—30% (*v/v*) AcN (final D_av_ = 25.8) led to UHPLC runs, which we consider as optimal for Spx separation (Figure 5G, P_sep_ = 28.7), whereas 85% (*v/v*) AcN (final D_av_ = 36.1, Figure 5H) resulted in an UHPLC run with lower P_sep_ (= 16.6) due to the reduced solubility of Spx in this solvent system.

In the preceding analysis, it could be shown for the *Sample* and *Syringe Solvent* that D_av_ has a lower limit of about 18, below which aggregation can occur, and an upper limit of about 30, above which Spx is less soluble. In addition, we have shown that Ac should be the main solvent component, because Spx is well-soluble in that solvent. A further improvement, i.e., a separation of the all-*trans* and *cis* peaks, is achieved, if additional compounds, like iPrOH or AcN, are used in the solvent mixture, which maybe interact more specifically with the Spx molecules. Here, AcN proved to be the best choice for this purpose.

### 3.2. Safety Considerations

The goal of this study was to find a UHPLC method for Spx, which would not only separate Spx from its biosynthetic precursors reliably, but would also exclude organic solvents, which are dangerous for both the experimentalist and the environment. Accordingly, we retained MeOH as the main component of the *Running Solvent* in the UHPLC system (since the *Running Solvent* is the solvent to be used in the highest volumes). MeOH has the advantage of being less volatile (vapor pressure at 20 °C: 13.0 kPa [33]) and less explosive than most of the other solvents considered (lower explosive limit: 6.0 vol.% [34]). In comparison, if very volatile solvents, such as Ac (vapor pressure: 24.5 kPa [33], lower explosive limit: 2.6 vol.% [34]) or MTBE (vapor pressure: 27.0 kPa [35], lower explosive limit: 1.6 vol.% [34]) are used in larger quantities (e.g., in the *Running Solvent*) for HPLC, their intense, pungent odors often cause headaches and nausea for many workers after a normal working day. Ac and MTBE exhibit a medium toxicity in the vapor phase (median lethal concentration (LC_50_) of Ac vapor: 31,500 ppm [34], and of MTBE: 23,600 ppm [34]). In contrast, MeOH vapor is approximately 3-fold less toxic than Ac and MTBE (i.e., essentially non-toxic: LC_50_ = 64,000 ppm [34]).

**Figure 8:**
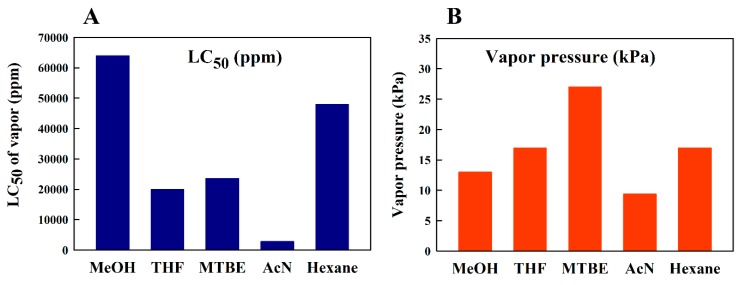
Comparison of (**A**) LC_50_ values and (**B**) vapor pressures of the solvents used here as *Running Solvent* (MeOH and THF) with solvents commonly used for HPLC separations and extraction procedures. The values shown were taken from [33,34,35]. The LC_50_ values have been obtained using the rat model, with the exception of AcN. For AcN, only data using the rabbit model are available in the public database.

The second component in the *Running Solvent* employed here, 2 vol.% THF, exhibits medium toxicity (vapor LC_50_ = 20,000 ppm [34]), and has a medium volatility (vapor pressure: 17.0 kPa [33], lower explosive limit: 1.8 vol.% [34]). However, THF also has an intensive odor, which serves to signal the experimentalist that more containment of the *Running Solvent* MeOH/THF (98/2 (*v/v*)) should be performed. This can be seen as an advantage compared to Hx, for example, which, though being equally volatile as THF (vapor pressure of Hx: 17.0 kPa [35]), exhibits only a subtle odor. We recognize that THF has potential disadvantages compared to the widely used MTBE: (i) THF can form peroxides during long-term storage, and (ii) exhibits a higher viscosity. The latter property may be disadvantageous for applications where gradients are employed, although the low amounts of THF present in our *Running Solvent* tend to offset these difficulties. For the solvents now commonly used for carotenoid extraction and HPLC separation, we have summarized the above discussion in Figure 8A,B. Figure 8A shows clearly the safety justification for using MeOH as a major component of the mobile phase, since it is about three-fold less toxic than commonly used second solvent components, such as THF or MTBE. The latter show almost equal toxicity, but Figure 8B shows that the volatility of MTBE is about one-third greater than that of THF. In typical biology laboratories, the higher MTBE volatility contributes significantly, and also noticeably to the vapor load. 

Note that we are also not suggesting that MTBE should now be dropped for the separation of carotenoids, since there may be many applications (e.g., separation of apocarotenoids) where MTBE could be superior as a separation solvent. However, for the application described here, the separation of well-known precursors of the carotenoid biosynthetic pathway starting with phytoene, we can see no advantage in using MTBE rather than THF.

In this study, we did not employ benzene, which had been shown to be a very good solvent for the separation of *cis* and all-*trans* isomers [11,12,13], but is highly carcinogenic [34]. Another commonly used solvent, DCM, is less carcinogenic than chloroform, but still exhibits a high toxicity (LC_50_ = 14,700 ppm [34] and is highly volatile (vapor pressure: 47.4 kPa [35]), though not explosive in air [34].

The widely-used solvent, AcN, is highly toxic (LC_50_ = 2850 ppm [34]) but has a lower vapor pressure (9.4 kPa [33]) than MeOH. However, AcN/air mixtures are highly explosive (lower explosive limit: 3.0 vol.% [34]). AcN is also a very aggressive solvent and will corrode plastic parts of the HPLC apparatus easily. Additionally, a comparison of the HPLC profiles presented here (Figure 6) with those obtained with AcN as main solvent [3,20,21], shows that a better peak separation and peak sharpening could be obtained with the *Running Solvent* MeOH/THF (98/2 (*v/v*)). Moreover, MeOH is less corrosive and much cheaper compared to AcN. An even more important point is that MeOH can be easily and rapidly degraded by methylotrophic bacteria, if it is accidentally released to the environment. The same is true for Ac (the main component of our *Sample* and *Syringe Solvent*) which is also easily biodegradable.

## 4. Materials and Methods

### 4.1. Chemicals and Carotenoid Sample Preparation

All organic solvents used were from Sigma-Aldrich/Merck (Darmstadt, Germany), AppliChem (Darmstadt, Germany), or Roth (Karlsruhe, Germany), and were of HPLC or LC/MS grade. Chemicals were from Sigma-Aldrich/Merck (Darmstadt, Germany). The ultrapure water used was taken from the Synergy^®^ UV water purification system from Millipore/Merck (Darmstadt, Germany).

The Spx and Anhdrvbr containing samples used in this study were isolated from the *R. rubrum* wild-type strain S1 (ATCC no. 11170, [36]). The lycopene-containing sample was isolated from the lycopene-producing *R. rubrum* mutant SLYC18 [23]. The *R. rubrum* cultures were grown anaerobically, photosynthetically in Sistrom minimal medium A (M medium, [37]), as described previously [38]. All experiments were performed at dim green light, and samples were gassed regularly with nitrogen to prevent oxidation. Cells were harvested by centrifugation (3000 × *g*, 10 min, 4 °C) and washed twice with 50 mM sodium-phosphate buffer, pH 7.0. The washed cell pellets were extracted twice with MeOH, in order to remove bacteriochlorophyll a and bacteriopheophytin, as well as quinones, which are highly soluble in MeOH (in contrast to Spx, see above). The resulting pellets were extracted with Hx, and the extracts gassed with N_2_ and stored at −80°C until further use.

The Spx-containing sample shown in Figure 2 and Figure 4 was obtained by purification of the Hx extract via a Sep-Pak^®^ column (Sep-Pak^®^ Vac 1cc (50 mg) C_18_ Cartridge, from Waters (Milford, CT, USA)), in order to remove residual lipids and proteins. For the Sep-Pak^®^ purification, 800 µL Hx extract was dried under N_2_ in a round-bottom flask and then dissolved in 1.5 mL MeOH/Hx (1/4 (*v/v*)). The dissolved sample was transferred to a 2 mL reagent tube and 0.5 mL MeOH added. The resulting suspension was pipetted onto the Sep-Pak^®^ column and pumped through the column using a rubber teat (note that after addition of the final MeOH aliquot, a phase separation occurs, which did not have any adverse effect upon the Sep-Pak^®^-purification). The resulting flow-through fraction was collected (fraction F1). The Sep-Pak^®^ column was washed with 10 mL Hx, and the Hx fraction W2 collected. The fractions F1 and W2 were dried with N_2_, dissolved in Hx, and stored at −80 °C (under N_2_) until further use. Both fractions were essentially free of protein or phospholipid contamination, as determined by TLC analysis (stationary phase: silica, mobile phase: pentane/ethanol (50/1 (*v/v*)) and could be injected into the UHPLC without further purification. In general, 50 µL of the F1-fraction were used for UHPLC. However, in Figure 4A, the peaks at 28 min and 33 min, respectively, indicate the presence of some residual bacteriochlorophyll precursors. These precursors are well separated from the carotenoids, and are thus unimportant for the analysis of carotenoid separation.

Nevertheless, for the systematic evaluation of the optimal solvent system, the sample to be analyzed was purified further by using a silica gel column (volume: 148 mL, height: 30 cm, diameter: 2.5 cm). The column was equilibrated with DCM, containing 1% (*v/v*) MeOH. The Spx- and Anhdrvbr-containing Hx extract (obtained from cells from an anaerobically, photosynthetically grown S1 culture) was dried by rotary evaporation at reduced pressure, dissolved in DCM/MeOH (99/1 (*v/v*)), and loaded onto the silica gel column. Elution was performed with the solvent combination DCM/MeOH (99/1 (*v/v*)). In the elution profile, Anhdrvbr was eluted first, well-separated from the last colored fraction which contained Spx. The fractions were dried by rotary evaporation, dissolved in Hx, and stored at −80 °C until further use. For UHPLC analysis, 10 µL and 5 µL of the concentrated Spx and Anhdrvbr fractions were used, respectively.

The lycopene-containing sample was obtained by purification of the Hx extract obtained from cells of a SLYC18 culture, via a Sep-Pak^®^ column as described above. For UHPLC analysis, 50 µL of the SepPak^®^-fraction W2 were employed. 

### 4.2. Carotenoid Sample Preparation for HPLC Runs

Immediately prior to the UHPLC experiment, the appropriate amount of carotenoid sample (usually 50 µL) was transferred to a 1.5 mL reagent tube. The sample was dried under N_2_, and then dissolved in 70 µL of the *Sample Solvent* by gentle mixing. After 5 min equilibration at room temperature, the carotenoid sample (usually now reduced to about 60 µL due to evaporation) was transferred to an HPLC vial with a glass insert for small volumes (Macherey-Nagel, Düren, Germany). The vial was placed into the autosampler, and the UHPLC run was started immediately, in order to prevent any precipitation of the sample in the vial. Then, 20 µL samples were injected into the sample loop via the UHPLC autosampler (see below).

### 4.3. UHPLC Setup and Settings

The HPLC column used was an ACE^®^ C_18_ column with 2 µm pore size (length: 150 mm, diameter: 4 mm) from VWR (Darmstadt, Germany). The UHPLC setup (UltiMate 3000 Series) was from Dionex^®^ (Thermo Fisher Scientific, Waltham, MA, USA) and consisted of a quaternary UHPLC pump (LPG-3400SD), an autosampler column compartment (ACC-3000T), and a variable wavelength detector (VWD-3100), which measured the absorbance at 480 nm (1 cm path-length in an analytical 11 µL flow cell) of the eluent continuously, after it had passed the column. Process control and analysis of the UHPLC system was performed using Chromeleon^TM^ (version 7.2 SR4) software. The temperature of the sample compartment and the column oven was set to 25 °C. The autosampler settings were as follows: inject 20 µL, full loop, wash volume: 1000 µL, wash speed: 50 µL/s. Even though the pump was equipped with its own degassing station, all solvents used were degassed by ultrasonification (in the ultrasonic bath SONOREX^TM^ RK 100 (Bandelin, Berlin, Germany)) for 7 min prior to use. The column was equilibrated with the isocratic *Running Solvent* MeOH/THF (98/2 (*v/v*)) using a reservoir connected to pump A, with a flow rate of 1 mL/min, prior to every experiment. The syringe was primed with the *Syringe Solvent* (see above) via a separate solvent reservoir connected to pump D. (Pump B and C were not in use, but were also filled with a solvent, which was mixable with the solvents used (e.g., 50% iPrOH (*v/v*) in H_2_O).) The pump rear seal was washed regularly (every 60 min) with 10% iPrOH (*v/v*) in H_2_O from an additional reservoir. Usually, a flow rate of 1 mL/min was used, which resulted in a pressure of about 160 bar. A typical UHPLC run lasted 45 min. Before and after every sample injection, a dummy run, where 20 µL of the *Sample Solvent* was injected (autosampler settings as above) and passed through the column was performed. This solvent blank run, which lasts 5 min at a flow rate of 1 mL/min, cleans the system and yields information about the height of the injection peak (at 1.5 min), which is different for each solvent.

For overnight or long-term storage, the syringe was primed with MeOH/THF (98/2 (*v/v*)), in order to remove the potentially corrosive solvents (e.g., AcN, MTBE, Ac). 

### 4.4. Calculation of D_av_

Generally, the dielectric constants (at 20 °C) of pure solvents were taken from the CRC Handbook of Chemistry and Physics [39]. However, the dielectric constants of solvents not listed in [39] i.e., AcN, THF, and MTBE, were taken from other reliable data sources [40,41,42]. The average dielectric constant of the solvent systems used was calculated as the sum of the individual dielectric constants D_s_ multiplied with their relative fractional coefficient σ_s_:

D_av_ = Σ_i_(σ_s_ × D_s_) (2)

where σ_s_ is the volume fraction of solvent S in the total solvent mixture.

### 4.5. Comparison of Solvent Toxicities

LC_50_ values were taken from the TOXNET databank from the NIH U.S. National Library of Medicine [34]. In general, the values obtained for rats (inhalation, 4 h) were used. For AcN, the LC_50_ value determined with rabbits (inhalation, 4 h) was used in the Discussion section, since no rat LC_50_ value was available. The LC_50_ value for DCM was obtained for rats after 6 h inhalation (a 4-h value was not available in the databank).

## 5. Conclusions

The highly conjugated, methoxylated C_40_ carotenoid, Spx, imposes a considerable challenge to finding a UHPLC method, which yields optimal peak resolution, while employing organic solvents which are environmentally friendly, providing minimal health risks, and as safe as possible for the researcher. Here, we have shown that the relatively harmless solvent combination MeOH/THF (98/2 (*v/v*)) can be used as *Running Solvent* for UHPLC chromatographic separation with optimal resolution. However, this solvent combination is only possible if an optimal delivery of the sample from the sample vial to the column is ensured. This is achieved by solubilizing the sample and filling the tubing system between sample vial and column with the *Sample* and *Syringe Solvent* Ac/AcN/iPrOH/MeOH (65/30/5/2 (*v/v/v/v*)).
